# Comparison of Knee Function After Reconstruction With Posterolateral Corner Injury and With or Without Posteromedial Corner Injury for Treating Knee Dislocation Cases: A Prospective Cohort Study

**DOI:** 10.1111/os.70277

**Published:** 2026-03-13

**Authors:** Yanlin Zhu, Hejun Sun, Xin Wang, Jian Li, Weili Fu

**Affiliations:** ^1^ West China Hospital of Sichuan University Chengdu Sichuan China; ^2^ Chengdu Seventh People's Hospital Chengdu Sichuan China; ^3^ Department of Joint and Sports Medicine, Tianjin Union Medical Center The First Affiliated Hospital of Nankai University Tianjin China

**Keywords:** ligament reconstruction ligament repair, medial collateral ligament, posterolateral corner injury, posteromedial corner injury

## Abstract

**Background:**

In the multiple ligament injury of the knee joint, apart from the anterior cruciate ligament and the posterior cruciate ligament, the Posterolateral Corner and the Posteromedial Corner are two structures that are easily overlooked. If not properly identified and repaired in one stage, the knee joint may be unstable, even failure of cruciate ligament reconstruction. The purpose of this article was to evaluate the effect of knee joint recovery after PLC (Posterolateral Corner) with or without PMC (Posteromedial Corner) injury.

**Methods:**

From 2016 to 2020, we screened a total of 2564 patients, of which 292 patients met the inclusion and exclusion criteria. In the end, a total of 44 people completed the study. Follow‐up was performed at 1, 3, 6, 9, and 12 months after surgery. We used pain visual analog scale (VAS) for pain, IKDC score, Lysholm score, Tegner score. Opti‐knee (a portable motion analysis system) was used to evaluate the stability of the knee joint at 1 year. In our prospective cohort study, we used the unpaired Student's *t*‐test for statistical analysis.

**Results:**

The knee joint function of PLC group and PLC combined PMC group was better than that before operation at 3 or 6 months after operation. Except for IKDC at 9‐month follow‐up and Tegner score at 9‐month and 12‐month follow‐up, there was no significant difference between the other groups.

**Conclusions:**

PLC and PLC combined with PMC injury showed similar prognostic effects, although the PLC group was numerically superior to the other group. We recommend primary repair and reconstruction in patients with confirmed PLC and PMC injuries to achieve the best postoperative recovery.

## Graphical abstract

This study assesses knee recovery after PLC/PLC+PMC injury repair: 44 patients followed up for 12 months, similar outcomes, PLC group numerically better; primary repair/reconstruction advised for optimal recovery.

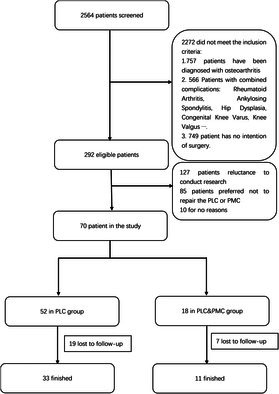

## 1 Introduction

Posterolateral Corner (PLC) injury usually consists of the following three parts: (1) lateral collateral ligament (LCL), (2) popliteofibular ligament (PFL), and (3) popliteal tendon (PT) [1, 2]. PLC is often referred to as the “dark corner” of the knee joint until the anatomy is properly recognized [3]. Isolated PLC injuries usually account for less than 30%, usually combined with anterior cruciate ligament (ACL) or posterior cruciate ligament (PCL) injuries [4, 5, 6, 7]. In the past, when the PLC function was not well understood, conservative treatment was usually the main method, which made the early diagnosis of grade III injury by Hughston classification not treated in time [8]. It will cause persistent lateral instability and even multidirectional instability of the knee joint, ultimately leading to the development of osteoarthritis and even failure of other ligamentous procedures that have been reconstructed [9, 10].

Therefore, for grade II and III injuries of PLC accompanied by other structural losses, the current literature is more inclined to use reconstruction methods for surgical treatment [11, 12]. There are many literature reports on the methods of reconstructing PLC, including: Larson's PLC reconstruction technique, LaPrade's posterolateral corner reconstruction technique, Arciero's posterolateral corner reconstruction technique, and Popliteal bypass reconstruction, etc. [13, 14, 15, 16]. All of these reconstructive procedures have been reported to have clinical effects on restoring the stability of the injured knee joint.

As mentioned above, PLC injuries are often accompanied by damage to other knee structures. Higher energy torsion or valgus and varus stress can cause damage not only to the PLC, but also to the Posteromedial Corner (PMC), another unnoticed anatomical location [17]. PMC lies between the posterior margin of the longitudinal fibers of the superficial medial collateral ligament (MCL) and the medial border of the PCL, comprises the superficial MCL, the deep MCL, the posterior oblique ligament (POL), the oblique popliteal ligament, and the posterior horn of the medial meniscus [18, 19, 20, 21]. It maintains the stability of the knee joint through the contraction mechanism of the semimembranosus [22]. Multiple structural injuries can make knee surgery challenging, and postoperative recovery may be uncertain. However, if PMC reconstruction or repair is not performed, it will lead to knee valgus and even rotational instability [23, 24]. This results in increased tension on the reconstructed ACL and PCL, which can eventually damage the knee.

At present, the literature has not reported the comparison of clinical effects after reconstruction of PLC injury and PLC combined with PMC injury. Therefore, we carried out this prospective cohort study. The purpose was to explore the preoperative and postoperative clinical improvement effect of the two reconstruction procedures and the difference in the improvement of knee joint function between the two procedures. We assumed that both reconstructions would achieve clinical improvement, but the effect of PLC injury reconstruction was due to the postoperative effect of PLC combined with PMC injury reconstruction.

## 2 Method

### 2.1 Study Design

We started our prospective cohort study in 2016 and registered at http://www.chictr.org.cn/index.aspx (ChiCTR‐OIC‐16008306). This study was approved by the Clinical Research and Biomedical Ethics Committee of West China Hospital, Sichuan University, with the ethics approval number: 2016‐97. We got the approval from the clinical research ethics committees. Each of the patients or otherwise their legal representative wrote the informed consent. Then we did the clinical trial in the West China hospital Sichuan University. This study followed the Good Clinical Practice guidelines and the guidelines of the Helsinki Declaration.

### 2.2 Criteria for Including, Excluding Patients

#### 2.2.1 Including Criteria

1. Patients with knee dislocation
2. PLC injury with two or more major ligaments (ACL, PCL, MCL, PMC) injured in knee joint shown by MRI.
3. Knee joint demonstrates obvious anterior/posterior and lateral/medial instability by physical examination.
4. No other severe physical injury that hinders limb functions.
5. Patients voluntarily agree to treatment protocol and are willing to cooperate in rehabilitation and follow‐up examination.

#### 2.2.2 Excluding Criteria

1. Patients with severe osteoarthritis.
2. Patients with combined complications which significantly hinder limb functions.
3. Patients that can't be treated or can't cooperate.

### 2.3 Participant

We enrolled patients at the Sports Medicine Center of West China Hospital between 2016 and 2020. Patients were identified by specialized sports medicine surgeons during routine outpatient consultations. The evaluation protocol encompassed medical history collection, physical examination, and imaging modalities (radiography, magnetic resonance imaging [MRI]). The study cohort was ultimately defined based on the aforementioned inclusion and exclusion criteria. Patients were stratified into two groups: the PLC injury group and the combined PLC and PMC injury group. Trained research personnel approached eligible patients to explain the study protocol and solicit their participation. Written informed consent was obtained from all participants prior to enrollment.

### 2.4 Operation Procedure

All operations are performed by two excellent surgeons. They lead all important steps in the surgery. There are no special requirements for surgical assistants.

#### 2.4.1 PLC Reconstruction

**FIGURE 1:**
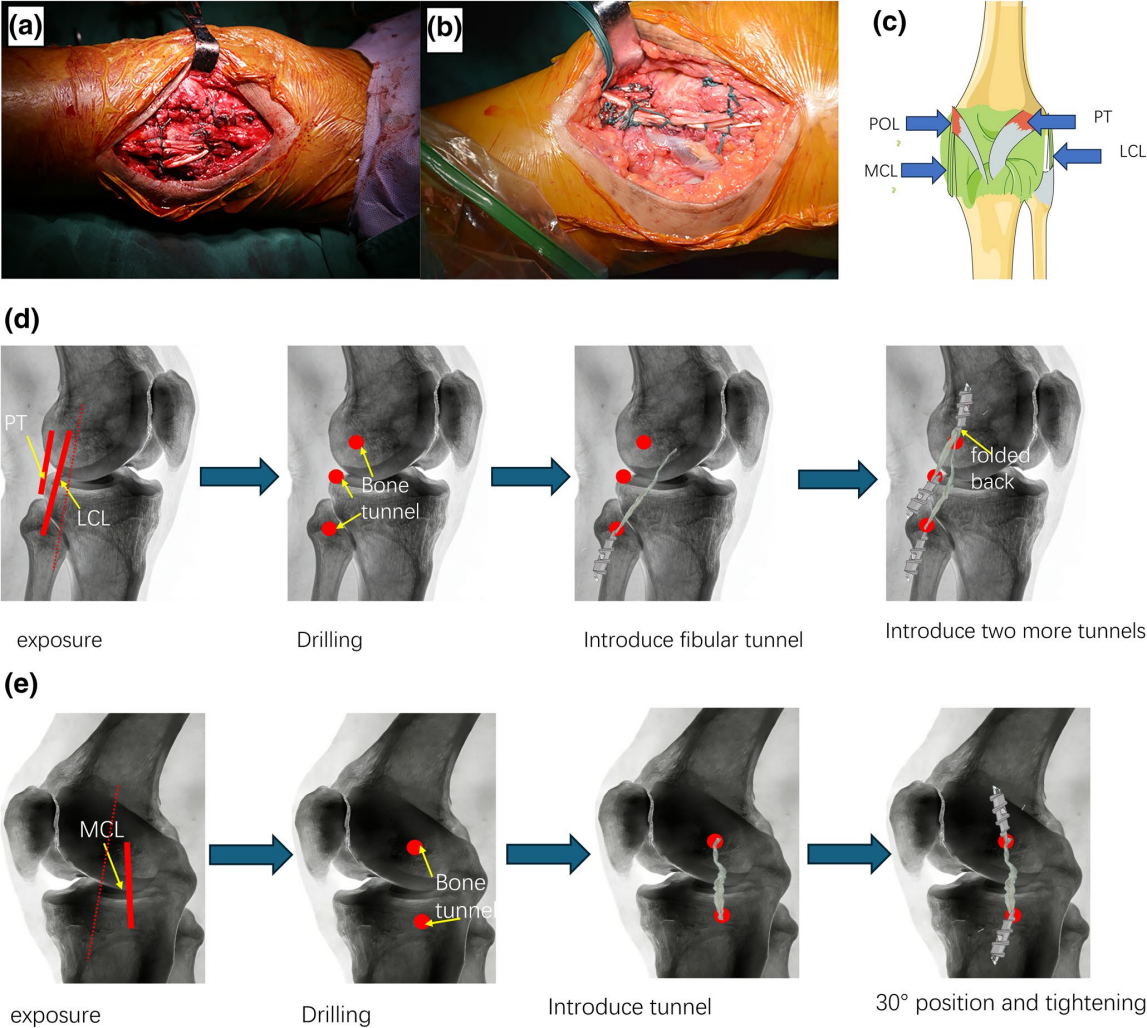
Surgery. (a, b) Intraoperative gross photographs. (c) Anatomical schematic diagram. (d, e) Simplified surgical procedural flowcharts.

Autologous hamstring tendon or peroneus longus tendon for PLC reconstruction surgery. The patient was placed in the supine position, the knee was flexed 90°, and the lateral epicondyle of the femur was taken as the turning point to make an incision on the lateral side of the knee joint. It runs proximally along the posterior border of the iliotibial band and distally to the plane of the fibular neck. Peel the flap forward and backward. From the inferior iliotibial band approach, the start point of the LCL and PT at the lateral femoral condyle is exposed medially, and the inferior border approach exposes the fibular start point of the LCL and the posterolateral ending point of the PT at the tibial plateau. A drill was used to establish bone tunnels at the LCL insertion of the lateral epicondyle of the femur, the LCL insertion of the fibula, and the posterolateral insertion of the PT tibial plateau. The tendon is first pulled into the fibular tunnel for compression fixation with absorbable screws. The tendon was then reflexed, and the reflex was introduced into the femoral tunnel, which was also squeezed with absorbable screws. Finally, the other end was introduced into the tibial tunnel, and compression fixation was performed under the condition of ensuring tension. Rinse the wound, suture the wound, and press the bandage (Figure 1a).

#### 2.4.2 PMC Reconstruction

The semitendinosus muscle was taken for PMC reconstruction. A longitudinal incision was made on the medial side of the knee joint, and a free flap was used to expose the start and ending point of the PMC: the lateral aspect of the medial condyle of the tibia and the lateral aspect of the medial femoral condyle. The establishment of the bone tunnel is carried out with a drill at the starting and ending points. Measure with the tendon so that the length of the tendon is slightly longer than the length between the two bone tunnels. Both ends of the tendon were introduced into the bone tunnel, and absorbable screws were used to slightly secure the tendon at the entrance of the bone tunnel. Tighten the screw with the knee flexed at 30° and under varus stress. The rest of the structure like POL is tightened and sutured on the outside with absorbable sutures or Transosseous suture. Rinse the wound, suture the wound, and press the bandage (Figure 1b). In the acute stage of PMC injury, when the MCL has not contracted or absorbed, anchor fixation and Transosseous suture are used to repair the MCL.

#### 2.4.3 ACL Reconstruction

For patients with concomitant ACL rupture, autologous hamstring tendon (semitendinosus and gracilis tendons) was used as the graft. The femoral tunnel was positioned at the anatomical insertion site (10–11 o'clock position for right knees, 1–2 o'clock for left knees) under arthroscopic guidance, and the tibial tunnel was placed at the center of the ACL footprint on the tibial eminence. Fixation was achieved with absorbable interference screws (femoral side) and adjustable cortical suspensory fixation (tibial side).

#### 2.4.4 PCL Reconstruction

Autologous Achilles tendon allograft was selected for PCL rupture. The femoral tunnel was drilled at the anatomical origin of the PCL (posteromedial aspect of the lateral femoral condyle), and the tibial tunnel was placed at the center of the PCL footprint on the posterior tibial plateau. Fixation was performed with absorbable interference screws (tibial side) and femoral cortical suspensory fixation.

#### 2.4.5 Timing of Concurrent Surgery

All ACL/PCL reconstructions were performed simultaneously with PLC/PMC repair/reconstruction in a single‐stage operation to avoid sequential surgical trauma and optimize joint stability recovery.

### 2.5 Postoperative Management

Postoperatively, a gradual rehabilitation principle of immobilization first, passive later, and active activity is adopted. A phased rehabilitation regimen was implemented postoperatively, adhering to the principle of initial immobilization, subsequent passive mobilization, and progressive active exercise, with specific milestones as follows:

- 0–2 weeks: The affected limb was immobilized in a brace at 0° of knee extension. Patients performed straight leg raise exercises and active quadriceps contraction training to maintain muscle strength without compromising surgical repair.
- 2 weeks: Gradual knee flexion training was initiated, with range of motion (ROM) progression tailored to individual healing status and surgical stability.
- 4 weeks: Brace restrictions were progressively loosened to permit increased active/passive knee motion while continuing ROM and light resistance training for periarticular muscles.
- 8 weeks: Strength and ROM exercises were intensified, including closed‐chain kinetic exercises (e.g., partial squats) and proprioceptive training to enhance joint stability.
- 3 months: The brace and crutches were discontinued. Patients commenced low‐intensity aerobic training (e.g., stationary cycling, walking) and were gradually reintegrated into normal daily activities, with return to higher‐demand activities deferred until 6 months based on clinical assessment.

### 2.6 Outcome Measures

Follow‐up was performed at 1, 3, 6, 9, and 12 months after surgery. We used the pain visual analog scale (VAS) for pain, IKDC score, Lysholm score, and Tegner score. Opti‐knee (a portable motion analysis system) was used to evaluate the stability of the knee joint at 1 year.

VAS is often used to assess pain. We use a 0–10 visual scale, with 0 representing no pain and 10 representing severe pain. Patients describe their pain by selecting numbers within this range [25]. The IKDC Subjective Knee Evaluation Form is scored by summing the scores of the individual items then transforming the score on the scale from 0 to 100 (by dividing the sum by 87, the total maximum possible score) then multiplying by 100. The higher the score, the higher the level of function and the lower the level of symptoms [26]. The Lysholm scale has been validated as a patient‐administered instrument to measure symptoms and function in patients with a variety of knee injuries. It measures the domains of symptoms and complaints and functioning in daily activities slightly. This scale consists of eight items. It is scored on a scale of 0 to 100, with higher scores indicating fewer symptoms and higher levels of functioning [27]. The Tegner score scores a person's activity level between 0 and 10 where 0 is ‘on sick leave/disability’ and 10 is ‘participation in competitive sports such as soccer at a national or international elite level’ [28]. Opti‐Knee (Innomotion Inc., Shanghai), was developed to track and analyze the 6DOF motion of the knee joint in a convenient and user‐friendly clinical setup. A digitizer was used to calibrate patient‐specific bone landmark points (i.e., great trochanter [GT], medial epicondyle [ME], lateral epicondyle [LE], medial tibia plateau [MP], lateral tibia plateau [LP], medial malleolus [MM], and lateral malleolus [LM]) with the participants in a neutral standing position. The neutral standing position was also used as a zero reference. Data about the positions of the marker sets was collected while the knee moved. The bony landmarks were calculated by the geometric relationship setup obtained from the initial position. 6DOF knee joint kinematics were calculated based on the local coordinate systems of the femur and the tibia using the bony landmarks. The averages and standard deviations of the knee kinematics from all gait cycles were calculated using an automated program [29].

### 2.7 Varus‐Valgus Angle

The maximum angular value of the knee joint in the varus‐valgus direction during the gait cycle (°), where positive values indicate valgus and negative values indicate varus.

### 2.8 Internal‐External Rotation Angle

The maximum angular value of the knee joint in the internal‐external rotational direction during the gait cycle (°), where positive values indicate external rotation and negative values indicate internal rotation.

### 2.9 Flexion‐Extension Angle

The maximum range of motion of the knee joint in flexion and extension during the gait cycle (°), which is the difference between the maximum flexion angle and the maximum extension angle.

### 2.10 Anterior–Posterior Displacement

The maximum anterior–posterior displacement distance of the tibia relative to the femur at the knee joint during the stance phase (cm), where positive values indicate anterior tibial translation and negative values indicate posterior tibial translation.

### 2.11 Superior–Inferior Displacement

The maximum superior–inferior displacement distance of the tibia relative to the femur at the knee joint during the gait cycle (cm), where positive values indicate superior tibial translation and negative values indicate inferior tibial translation.

### 2.12 Internal‐External Displacement

**FIGURE 2:**
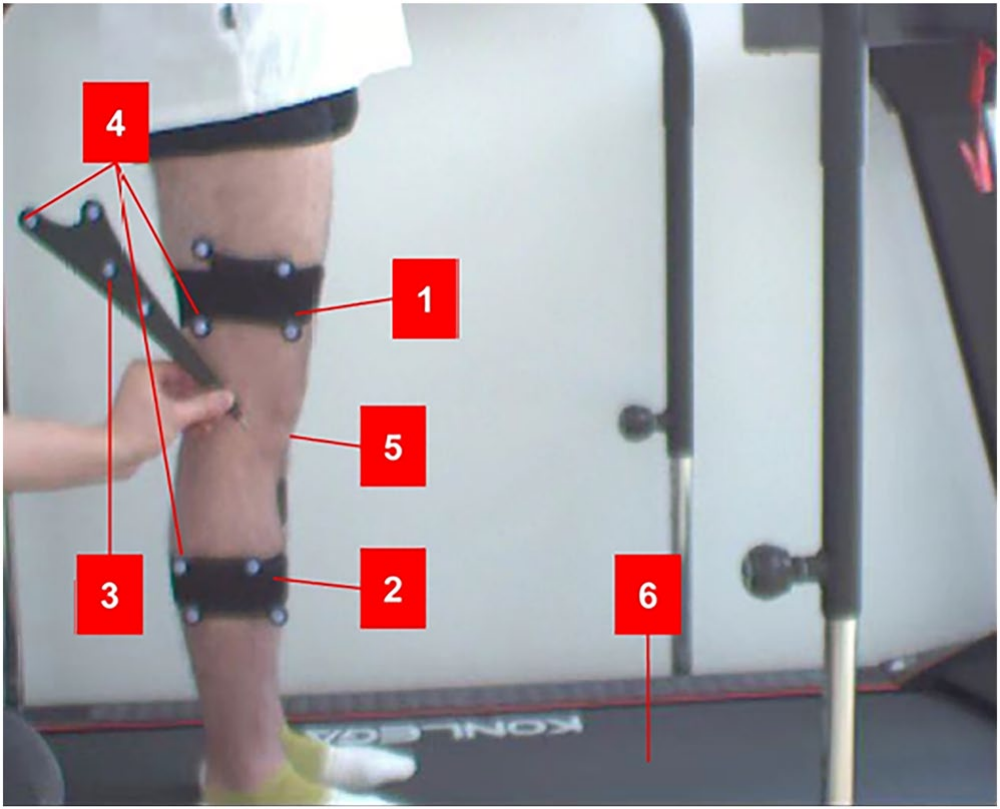
The perspective of high‐speed camera in opti‐knee. 1. Combined cursor on femur; 2. Combined cursor on tibia; 3. A hand‐held probe; 4. Spatial coordinates determined by a combination of femoral and tibial cursors and the hand‐held probe.; 5. Marked surface anatomical landmarks on the femur lateral condyle; 6. Treadmill.

The maximum medial‐lateral displacement distance of the tibia relative to the femur at the knee joint during the stance phase (cm), where positive values indicate medial tibial translation and negative values indicate lateral tibial translation (Figure 2).

### 2.13 Statistical Analysis

Continuous variables were presented as mean ± standard deviation (SD), and categorical variables were reported as frequencies (*n*). Comparisons of the Visual Analogue Scale (VAS), International Knee Documentation Committee (IKDC) score, Lysholm score, and Tegner activity score between the two groups at each preoperative and postoperative follow‐up time point were performed using the unpaired Student's *t*‐test. Descriptive statistics (mean ± SD) for kinematic parameters were additionally generated using the built‐in analytical module of Opti‐knee software. A two‐tailed *p*‐value < 0.05 was considered statistically significant. All statistical analyses were conducted using IBM SPSS Statistics for Windows, Version 18.0 (IBM Corp., Armonk, NY, USA).

A post hoc power analysis was performed using G*Power 3.1 software to evaluate the statistical power of the study. Taking the 12‐month Lysholm score (the primary outcome measure) as the core indicator, the analysis was conducted with a significance level (*α*) set at 0.05 and a type II error rate (*β*) set at 0.20. The results showed that the statistical power of this study was 0.72, which meets the minimum power requirement (≥ 0.70) for clinical cohort studies, indicating that the sample size is sufficiently powered to detect meaningful differences in the primary outcome between the two groups. The minimum sample size required per group was 30 patients for the PLC injury group and 10 patients for the combined PLC‐PMC injury group. This pre‐calculated threshold was used to guide patient enrollment, and the final analyzed cohort (33 in PLC group, 11 in PLC‐PMC group) exceeded the minimum requirement to ensure statistical robustness.

## 3 Result

### 3.1 Basic Characteristics

A total of 2564 patients were screened at the Sports Medicine Center of West China Hospital between 2016 and 2020, among whom 292 individuals met the predefined inclusion and exclusion criteria. The reasons for exclusion were categorized as follows:

- 757 patients had a current or previous diagnosis of knee osteoarthritis, which was deemed a confounding factor for functional outcome assessment;
- 566 patients suffered from severe comorbidities that could affect lower extremity motor function or weight‐bearing capacity, including rheumatoid arthritis, knee varus deformity, and other orthopedic or rheumatologic disorders;
- 749 patients were excluded due to either mild disease severity (not warranting surgical intervention) or economic constraints precluding participation in the study protocol.

**FIGURE 3:**
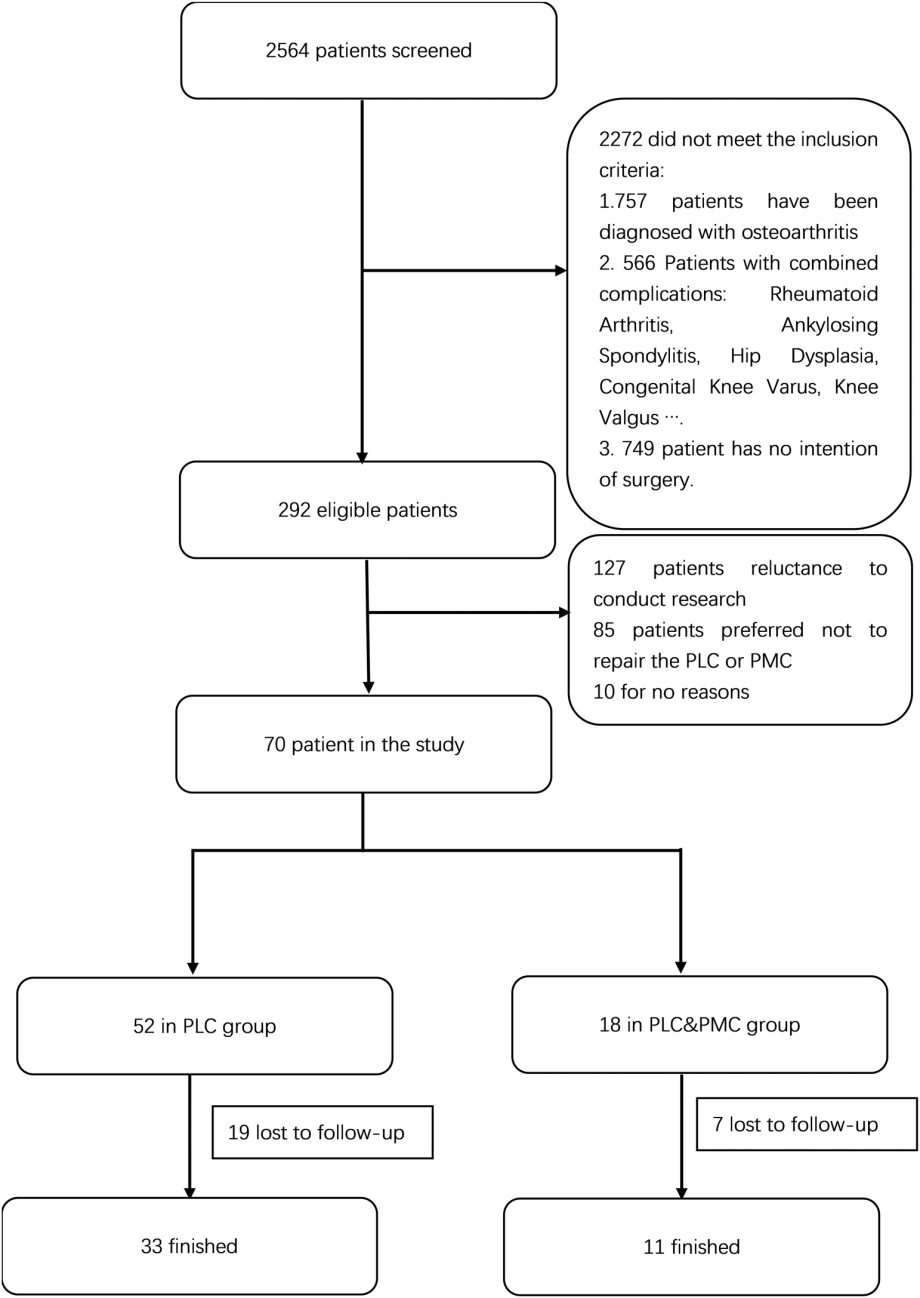
Flowchart of patient screening.

Ultimately, 70 eligible patients were enrolled in the study, with 52 grouped to the PLC injury group and 18 to the combined PLC and PMC injury group. During the follow‐up period, 19 patients in the PLC group and 7 patients in the PLC&PMC group were lost to follow‐up due to incomplete contact information or withdrawal of consent. A total of 44 patients (33 from the PLC group and 11 from the PLC&PMC group) completed the entire study protocol and were included in the final analysis. All details are shown in Figure 3.

Among patients who completed the study, the PLC injury group (*n* = 33) had a male‐to‐female ratio of 22:11, while the combined PLC and PMC injury group (*n* = 11) had a male‐to‐female ratio of 8:3; the mean age was 44.4 years (SD = 12.25) in the PLC group and 36.8 years (SD = 9.82) in the PLC&PMC group. Preoperative functional scores showed no statistically significant differences between groups: IKDC score (PLC group 27.52 ± 14.64 vs. PLC&PMC group 25.22 ± 12.85, *p* > 0.05), Lysholm score (PLC group 29.76 ± 21.58 vs. PLC&PMC group 33.90 ± 21.49, *p* > 0.05), and Tegner activity score (PLC group 0.59 ± 1.10 vs. PLC&PMC group 0.73 ± 0.96, *p* > 0.05). In contrast, the preoperative VAS for pain exhibited a significant between‐group difference (PLC group 7.47 ± 2.42 vs. PLC&PMC group 4.80 ± 2.71, *p* < 0.05). In the PLC group, 28 cases (84.8%) were complicated with PCL injury and 15 cases (45.5%) with ACL injury. In the PLC + PMC combined reconstruction group, 9 cases (81.8%) were complicated with PCL injury and 8 cases (72.7%) with ACL injury. There was no statistically significant difference in the distribution of combined ligament injuries between the two groups (*p* > 0.05). All patients achieved primary wound healing postoperatively, with no major complications (e.g., infection, neurovascular injury, or graft failure) reported during the follow‐up period. The baseline characteristics of the researchers are shown in Table 1.

**TABLE 1:** Baseline characteristics of the study population.

	PLC	PLC&PMC
Gender (male: female)	22:11	8:3
Age	44.4 ± 12.25	36.8 ± 9.82
Time to surgery (days)	15	14
BMI	21 ± 13	20.7 ± 12
IKDC	27.52 ± 14.64	25.22 ± 12.85
Lysholm	29.76 ± 21.58	33.9 ± 21.49
Tegner	0.586 ± 1.10	0.73 ± 0.96
VAS	7.47 ± 2.42	4.8 ± 2.71
Complications	0	0
ACL injured	15	9
PCL injured	28	8

### 3.2 Patient Reported Outcome Measures

With respect to the IKDC score, no statistically significant difference was observed between the PLC injury group and the combined PLC and PMC injury group prior to surgery. At the 1‐ and 3‐month follow‐up time points, IKDC scores in both groups were lower than the preoperative baseline levels, while at the 6‐month follow‐up, scores in both groups initially exceeded the preoperative values—a trend that reached statistical significance by the 9‐month follow‐up. Notably, between‐group comparisons revealed a statistically significant difference only at the 9‐month follow‐up (PLC group: superior to PLC&PMC group, *p* = 0.03), with no significant discrepancies detected at all other follow‐up time points. The detail information shown in Table 2.

**TABLE 2:** Patient reported outcome measures.

	PLC	PLC&PMC	*p*
IKDC			
Preoperative	27.52 ± 14.64	25.22 ± 12.85	0.62
1 month	17.07 ± 7.46	11.85 ± 6.73	0.03
3 months	24.18 ± 9.42	23.20 ± 5.62	0.68
6 months	40.11 ± 13.25	34.62 ± 10.51	0.16
9 months	53.39 ± 11.80	43.31 ± 13.57	0.03
12 months	63.60 ± 11.17	55.19 ± 18.03	0.15
Lysholm			
Preoperative	29.76 ± 21.58	33.9 ± 21.49	0.58
1 month	29.5 ± 15.84	29.09 ± 13.78	0.93
3 months	48.2 ± 17.81	55.6 ± 23.44	0.34
6 months	64.13 ± 16.91	62.5 ± 20.98	0.82
9 months	72.13 ± 15.49	66.9 ± 16.32	0.35
12 months	82.39 ± 16.46	73.56 ± 17.75	0.15
Tegner			
Preoperative	0.586 ± 1.10	0.73 ± 0.96	0.68
1 month	0.32 ± 0.614	0.45 ± 0.66	0.67
3 months	1.25 ± 0.99	1.40 ± 0.66	0.57
6 months	2.05 ± 1.02	1.75 ± 0.83	0.33
9 months	3.06 ± 1.00	2.3 ± 1.0	0.03
12 months	3.64 ± 1.20	2.78 ± 1.03	0.02
VAS			
Preoperative	7.47 ± 2.42	4.8 ± 2.71	0.0004
1 month	6.42 ± 2.15	5.18 ± 2.03	0.08
3 months	4.65 ± 1.49	4.1 ± 1.51	0.29
6 months	4 ± 1.31	3.63 ± 1.11	0.36
9 months	3.12 ± 1.08	3.3 ± 0.78	0.55
12 months	2.27 ± 0.76	2 ± 0.67	0.98

For the Lysholm score, no statistically significant differences were observed between the PLC injury group and the combined PLC and PMC injury group at all preoperative and postoperative follow‐up time points; both groups exhibited lower scores at 1 month postoperatively compared with preoperative levels, but by 3 months postoperatively, scores had improved to surpass preoperative values with statistical significance. Regarding the Tegner activity score, a similar recovery trend was noted—scores in both groups were lower at 1 month postoperatively than preoperatively, then significantly improved to exceed baseline by 3 months, and intergroup comparison revealed a statistically significant difference starting from the 9‐month follow‐up, with the PLC group demonstrating superior outcomes. For the VAS score, a significant preoperative difference existed between the two groups, but this discrepancy resolved after surgery, with no significant intergroup differences observed at any postoperative follow‐up time point. As for the IKDC score, no preoperative intergroup difference was found; scores in both groups remained lower than baseline at 1 and 3 months postoperatively, began to exceed preoperative levels by 6 months, and reached statistical significance by 9 months, with the only significant intergroup difference occurring at the 9‐month follow‐up (PLC group superior to PLC&PMC group, *p* = 0.03).

### 3.3 Opti‐Knee Outcome

At 1 year postoperatively, the Opti‐knee system was utilized to evaluate the knee joint stability of the affected limb, encompassing six key parameters: varus‐valgus angle, internal‐external rotation, flexion‐extension angle, anterior–posterior displacement, superior–inferior displacement, and internal–external displacement. Detailed measurements for these parameters are presented in Table 3. Horizontal comparisons of the six stability indices revealed that the PLC injury group exhibited numerically higher knee joint stability than the combined PLC and PMC injury group, though this difference did not reach statistical significance (all *p* > 0.05).

**TABLE 3:** Opti‐knee outcome at 1 year follow‐up.

	PLC		PLC&PMC		*p*
	Mean	SD	Mean	SD	
Varus and valgus angles (°)	5.1	14.43	9.66	19.05	0.48
Internal and external rotation (°)	6.01	15.58	10.58	18.42	0.46
Flexion and extension angle (°)	34.57	66.95	49.53	65.33	0.51
Forward and backward displacement (cm)	0.86	2.33	1.33	2.57	0.59
Up and down displacement (cm)	1.13	2.5	1.27	2.23	0.86
Internal and external displacement (cm)	0.59	1.65	0.46	1.35	0.79

**FIGURE 4:**
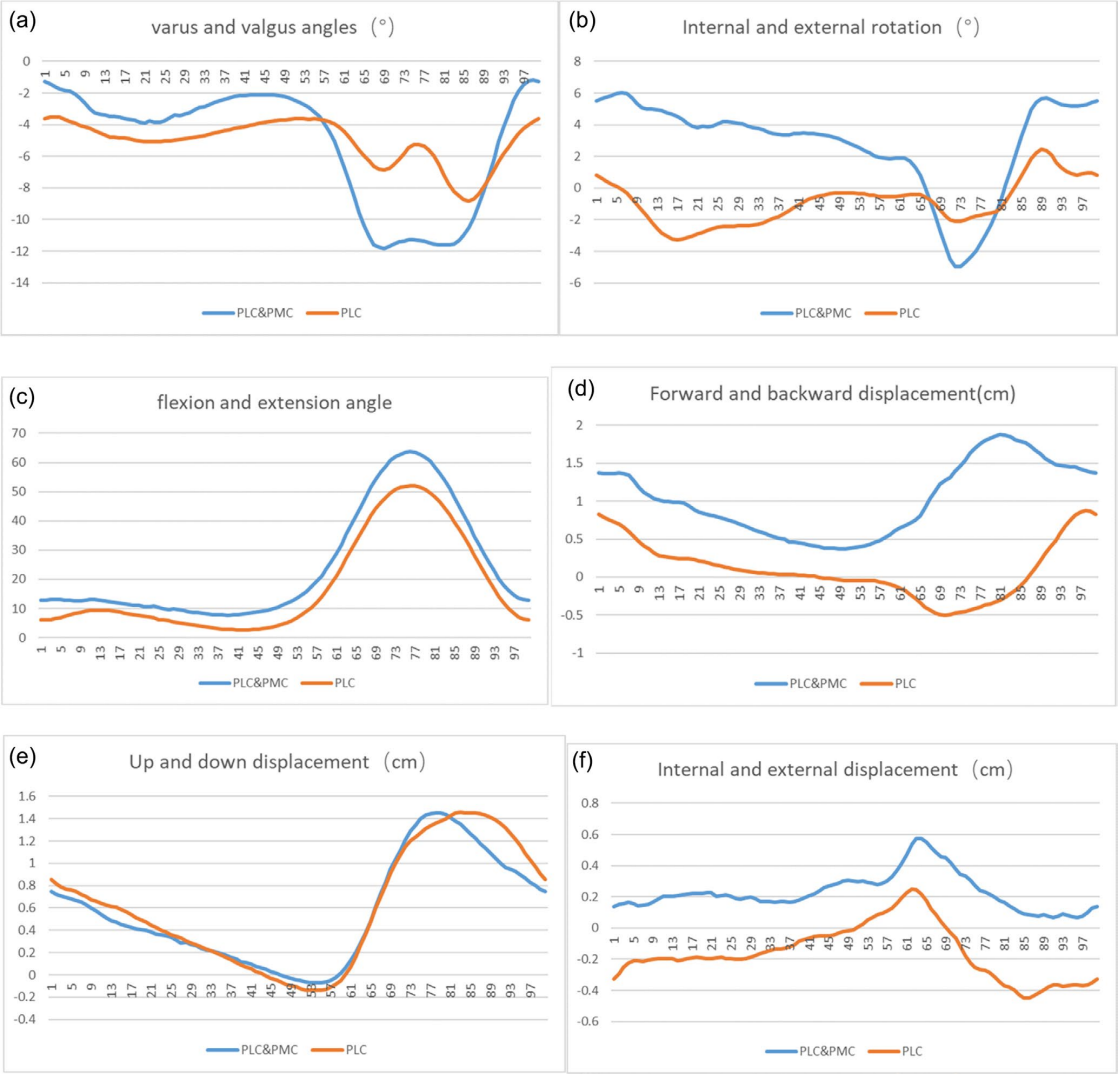
Between PLC and PLC&PMC: (a) varus and valgus angles (°), (b) internal and external rotation (°), (c) flexion and extension angle, (d) forward and backward displacement (cm), (e) up and down displacement (cm), and (f) internal and external displacement (cm).

To further characterize dynamic stability, we summarized the performance of the six parameters across a single gait cycle and generated corresponding quantitative curves (Figure 4a–f). In these curves, the ordinate denotes the magnitude of parameter change (with positive and negative values indicating opposite directions of displacement or angular variation), while the abscissa represents the phase within the gait cycle. Dynamic analysis demonstrated consistent valgus and anterior instability patterns in both groups; notably, the PLC group displayed relatively superior stability throughout the gait cycle, yet this intergroup discrepancy remained statistically non‐significant (*p* > 0.05).

Primary wound healing was achieved in all cases postoperatively, with no major complications (e.g., infection, neurovascular compromise, or graft failure) reported during the entire follow‐up.

## 4 Discussion

### 4.1 Main Finding

Preliminary evidence shows combined PLC‐PMC injuries may achieve prognostic outcomes similar to isolated PLC injuries after primary repair and reconstruction (supporting active surgical intervention for complex cases), and primary repair/reconstruction is recommended for confirmed PLC/PMC injuries to reduce long‐term complications, while extending postoperative motor function training cycles for combined injury patients may optimize recovery. In this prospective cohort study, we investigated the prognostic effect between two groups after PLC and PLC combined with PMC injury surgery. The purpose is to explore the effectiveness of surgery. We included 44 patients, followed up for 1 year, and assessed postoperative outcomes in terms of scoring scales and objective range of motion of the knee joint. The results showed that, except for the IKDC score at the 9‐month follow‐up time point and the Tegner score between the two groups at the 9‐ and 12‐month follow‐up time points, there was no significant statistical difference in all the score scale data at the other follow‐up time. The data in both groups were better than the preoperative score at the 3‐ or 6‐month follow‐up time point and finally reached a statistical difference. The stability of PLC in the Opti‐knee evaluation was better than that of PLC&PMC, but there was no statistical difference in the data of six aspects.

### 4.2 Comparison of Conclusions With Similar Studies

The reconstruction method we adopt is more similar to Larson's reconstruction method, namely the fibular‐based method [13]. This technique was shown to be effective in both varus and external rotation of the knee joint following a cadaver‐based biomechanical study by Wang et al. and liu et al. [29, 30]. Although there are many methods for PLC reconstruction, according to the existing literature reports, the mainstream reconstruction methods are also effective in restoring the stability of the knee joint [31, 32] and there is still no consensus about which technique is better for reconstructions [33, 34]. Therefore, we do not have special regulations on the surgical method, as long as the final result can reconstruct the PLC structure.

A core finding of this study is the comparable overall surgical efficacy between the two groups, despite differences in injury complexity. Both groups demonstrated a consistent trajectory of functional recovery: scores were transiently lower at 1 month postoperatively (likely due to surgical trauma and early rehabilitation constraints) but rebounded to exceed preoperative baselines by 3–6 months, with statistically significant improvements sustained through the 12‐month follow‐up. Intergroup differences were only observed in the IKDC score (9‐month follow‐up) and Tegner score (9‐ and 12‐month follow‐ups), where the isolated PLC group exhibited superior outcomes. This nuanced difference aligns with the “injury severity gradient”—combined PLC‐PMC injury involves more extensive soft tissue disruption, leading to slower recovery of activity levels (reflected in Tegner scores) and functional performance (reflected in IKDC scores). Importantly, however, these differences did not translate to meaningful clinical disparities in pain relief (VAS) or overall knee function (Lysholm), confirming that combined injuries can still achieve satisfactory outcomes with appropriate surgical intervention.

Objective stability assessment via the Opti‐knee system further supplemented these findings. While the isolated PLC group showed numerically better performance across all six stability parameters (varus‐valgus angle, internal‐external rotation, flexion‐extension angle, and three displacement dimensions), no statistical differences were detected [35]. The dynamic gait cycle curves (Figures 2, 3, 4) provided additional granularity, revealing that both groups exhibited valgus and anterior instability—a common residual deficit after posterolateral ligamentous repair—but the PLC group maintained marginally better stability throughout the gait phase. This numerical advantage may be attributed to the preserved PMC integrity in the isolated PLC group, as the PMC (particularly the posterior oblique ligament, POL) plays a critical role in resisting valgus and rotational forces [34]. The absence of statistical significance, however, underscores the effectiveness of our one‐stage repair strategy for combined injuries, which included MCL reconstruction and POL fixation (via screws or sutures) to minimize bone tunnel interference and restore key stabilizing structures [36].

In PMC repair and reconstruction, Kim et al. showed that after simultaneous MCL and POL reconstruction, the patient recovered well with a Lysholm score of 91.9 at 52.6 months [37]. In the study of Kitamura et al., only the MCL structure was reconstructed, and a good clinical cure effect could be achieved after surgery [38]. At present, in a setting of a multi‐ligament knee injury involving the PMC, early concurrent augmentation (if the posterior oblique ligament can be repaired) is recommended to facilitate early mobilization and rehabilitation [39]. In the operation of this study, in order to reduce the interference between the bone tunnels, the MCL was reconstructed, and the POL structure was screwed or sutured to achieve better clinical results [40].

We can see that the VAS score is better than PLC&PMC in preoperative PLC, which may be because the latter damages more knee joint structures in the process of injury, which makes the pain more severe. There was no statistically significant difference in pain between the two groups after surgery, which can be understood as the two types of surgery can relieve the pain of the patients, and the effect is similar [41]. The more severe the injury, the longer the post‐operative functional recovery may take, and the less rapid the recovery; and vice versa. This may also be the result of a statistically significant difference in Tegner scores at the 9‐ and 12‐month follow‐up time points [42]. The remaining results did not show statistical differences between the groups, indicating that the clinical effects were similar between the two groups [36].

Due to the lack of current research on PMC, there are still different opinions in the current literature on its components. The lack of understanding of it has led to omissions in the repair and reconstruction process. The current PMC reconstruction technology is more anatomical reconstruction along the course of the MCL, while ignoring the repair of other components, which is also used in this paper. The currently recognized biomechanical mechanisms of PMC are Semimembranosus Tendon and Expansions. It consists of five major arms: (1) the reflex (forearm), (2) direct medial tibial insertion (primary attachment), (3) OPL insertion, (4) POL insertion, and (5) popliteal aponeurosis expansion [43]. Damage to any one structure can lead to an imbalance in other parts during the contraction process. This eventually results in muscle spasms and knee instability [44]. The POL has the same stability as the MCL in maintaining selective stability of the knee, and neglecting the POL structure during surgery can lead to rotational instability of the knee [45, 46]. This may be the reason why the rotational stability of the PLC&PMC group was numerically inferior to that of the PLC group after surgery in this study. However, there was no statistical difference between the groups, which makes us believe that one‐stage PMC repair and reconstruction still has clinical value.

### 4.3 Strengths and Limitations

Despite the strengths of this study—including its prospective cohort design, standardized surgical and rehabilitation protocols, objective knee stability assessment via the Opti‐knee system, and provision of short‐term (1‐year) prognostic references for primary repair/reconstruction in patients with confirmed PLC injuries (with or without concomitant PMC and other major ligament injuries)—it still has several limitations: first, the relatively small final sample size (44 patients completing follow‐up), which is prone to scale measurement and statistical bias, stemming from the low clinical incidence of combined PLC‐PMC injuries (accounting for 5%–8% of all knee ligament injuries) and stringent inclusion criteria (requiring MRI‐confirmed multi‐ligament injuries, significant clinical instability, specified concurrent surgeries, and 1‐year multi‐point follow‐up), leading to exclusion of potential cases due to mild injuries, financial constraints, or refusal of long‐term follow‐up; second, although the Opti‐knee system facilitated in‐depth understanding of postoperative knee stability, data deviation may occur due to positioning point issues, necessitating further training for investigators; third, the choice of surgical method may substantially impact prognosis, and future surgeries should include more detailed repair of secondary structures beyond primary ones. Consequently, the conclusions of this study are primarily applicable to the aforementioned patient population, and future research should expand the sample size through multi‐center collaborations and extend the follow‐up period to 3–5 years to further verify the long‐term efficacy and safety of primary repair/reconstruction, thereby enhancing the generalizability and clinical reference value of the findings.

## 5 Conclusion

### 5.1 Main Findings

1. Primary repair and reconstruction yielded notable improvements in knee function (pain, motor function, joint stability) for both isolated PLC and combined PLC‐PMC injuries; recovery accelerated at 3–6 months postoperatively, with satisfactory outcomes at 12 months.
2. No statistically significant intergroup differences were found in postoperative VAS (pain), Lysholm (overall function), or Opti‐knee‐derived stability parameters.
3. Group disparities were restricted to specific indicators: the isolated PLC group showed higher IKDC scores at 9 months and Tegner scores at 9–12 months postoperatively. This implies that more extensive soft tissue damage in combined injuries may delay motor function recovery slightly, while final clinical efficacy remained comparable between groups.

### 5.2 Clinical Implications

1. Preliminary evidence suggests combined PLC‐PMC injuries may achieve prognostic outcomes similar to isolated PLC injuries after primary repair and reconstruction, providing tentative evidence‐based support for active surgical intervention in such complex cases.
2. Primary repair and reconstruction are recommended for confirmed PLC and PMC injuries to potentially reduce long‐term complications like joint instability and cruciate ligament reconstruction failure linked to missed repair.
3. For combined injury patients, extending postoperative motor function training cycles may help optimize functional recovery, offering a reference for clinical rehabilitation planning.

## Author Contributions

All authors contributed to the study conception and design. Material preparation, data collection, and analysis were performed by Yanlin Zhu. The first draft of the manuscript was written by Yanlin Zhu and all authors commented on previous versions of the manuscript. All authors read and approved the final manuscript.

## Funding

The authors have nothing to report.

## Disclosure

The authors affirm that human research participants provided informed consent for publication of the images in Figure 4e.

## Ethics Statement

We started our prospective cohort study in 2016 and registered at http://www.chictr.org.cn/index.aspx (ChiCTR‐OIC‐16008306). We got the approval by the clinical research ethics committees. Each of the patients or their legal representatives wrote the informed consent. This study was approved by the Clinical Research and Biomedical Ethics Committee of West China Hospital, Sichuan University, with the ethics approval number: 2016‐97. Then we did the clinical trial in the West China hospital Sichuan University. This study followed the Good Clinical Practice guidelines and the guidelines of the Helsinki Declaration.

## Consent

Informed consent was obtained from all individual participants included in the study.

## Conflicts of Interest

The authors declare no conflicts of interest.

## Supporting information


**Table S1:** Baseline characteristics of all population.

**Table S2:** Attrition Reasons.

## Data Availability

The data that support the findings of this study are openly available in Pubmed at https://pubmed.ncbi.nlm.nih.gov/.
