# Supplementary material for: Comparison of Knee Function After Reconstruction With Posterolateral Corner Injury and With or Without Posteromedial Corner Injury for Treating Knee Dislocation Cases: A Prospective Cohort Study
Source: Orthop Surg. 2026 Mar 13;18(4):721–32. doi: 10.1111/os.70277 (PMC13056492; doi:10.1111/os.70277)
Supplement: Supplementary file 1 — Table S1: Baseline characteristics of all population. [file OS-18-721-s001.docx]

|  | PLC | PLC&PMC | Loss |
| --- | --- | --- | --- |
| Gender (male: female) | 22:11 | 8：3 | 26 |
| age | 44.4±12.25 | 36.8±9.82 | 40.6±11.04 |
| Time to surgery(days) | 15 | 14 | / |
| BMI | 21±13 | 20.7±12 | 20.85±12.5 |
| IKDC | 27.52±14.64 | 25.22±12.85 | 26.37±13.75 |
| Lysholm | 29.76±21.58 | 33.9±21.49 | 31.83±21.54 |
| Tegner | 0.586±1.10 | 0.73±0.96 | 0.658±1.03 |
| VAS | 7.47±2.42 | 4.8±2.71 | 6.14±2.57 |
| ACL injured | 15 | 9 | 12 |
| PCL injured | 28 | 8 | 10 |

Table s1 **Baseline characteristics of all population**
