# Supplementary material for: Comparison of Knee Function After Reconstruction With Posterolateral Corner Injury and With or Without Posteromedial Corner Injury for Treating Knee Dislocation Cases: A Prospective Cohort Study
Source: Orthop Surg. 2026 Mar 13;18(4):721–32. doi: 10.1111/os.70277 (PMC13056492; doi:10.1111/os.70277)
Supplement: Supplementary file 2 — Table S2: Attrition Reasons. [file OS-18-721-s002.docx]

| Attrition Reasons | PLC Injury Group (n=19) | PLC+PMC Injury Group (n=7) | Total (n=26) |
| --- | --- | --- | --- |
| Unreachable (lost contact) | 8 | 3 | 11 |
| Voluntary withdrawal of informed consent | 4 | 2 | 6 |
| Relocation to other cities (out of follow-up area) | 3 | 1 | 4 |
| Unable to continue due to new-onset medical conditions | 2 | 0 | 2 |
| Financial burden of rehabilitation and reexamination | 1 | 0 | 1 |
| Time constraints (work/study conflicts) | 1 | 1 | 2 |
| Dissatisfaction with initial rehabilitation progress | 0 | 0 | 0 |
| Total | 19 | 7 | 26 |

Table s2 Attrition Reasons
